# Alzheimer’s Association Interdisciplinary Summer Research Institute (AA-ISRI): Insights From Program Alumni

**DOI:** 10.1093/geroni/igaf122.1507

**Published:** 2025-12-31

**Authors:** Andrew Pickett, Quinton Cotton, Emily Mroz, Kalisha Bonds Johnson, Manka Nkimbeng

**Affiliations:** Indiana University School of Public Health- Bloomington, Bloomington, Indiana, United States; University of Pittsburgh, Pittsburgh, Pennsylvania, United States; Emory University, Atlanta, Georgia, United States; Emory University, Atlanta, Georgia, United States; University of Minnesota School of Public Health, Minneapolis, Minnesota, United States

## Abstract

Held annually since 2021, the Alzheimer’s Association Interdisciplinary Summer Research Institute (AA-ISRI) provides an immersive, no-cost professional development opportunity for dementia researchers. The week-long institute provides training on innovations in dementia science, offers peer group support and networking, and structured mentorship centered around grant writing and career coaching to ensure success in a research career. Organized across two overarching research tracks (i.e., psychosocial, public health) and by participant interests, daily peer group meetings (4-6 people) are led by established mentors. Presenters in this session have all completed the AA-ISRI program and will share experiences and insights related to participation. Presenters’ experiences broadly aligned with three overarching themes: (1) an interdisciplinary approach to dementia research training, (2) emphasis on peer and mentorship networking, and (3) opportunities to interact with field-leaders and funding agency representatives. AA-ISRI, by definition, is interdisciplinary—accepting participants from a variety of educational backgrounds (e.g., MD, PhD) and experiences (e.g., public health, nursing, social work, medicine). As such, the program curriculum introduces the state-of-the-science across multiple subdisciplines within the wider field of dementia research (e.g., pathology/ treatment, trial design, participant recruitment, implementation science). In small group meetings, participants receive feedback on an in-progress grant proposal and learn to develop an effective ‘elevator pitch’ for their research. Finally, each participant has one-on-one mentorship meetings with leading experts in their area and opportunities to interact with research funders. Thus, AA-ISRI provides an impactful opportunity for early career and new-to-the-field investigators, as well as more established researchers transitioning into dementia research.

